# Vitamin D Prevents Hypoxia/Reoxygenation-Induced Blood-Brain Barrier Disruption via Vitamin D Receptor-Mediated NF-*k*B Signaling Pathways

**DOI:** 10.1371/journal.pone.0122821

**Published:** 2015-03-27

**Authors:** Soonmi Won, Iqbal Sayeed, Bethany L. Peterson, Bushra Wali, Jared S. Kahn, Donald G. Stein

**Affiliations:** Department of Emergency Medicine Brain Research Laboratory, Emory University, Atlanta, Georgia, United States of America; Washington University, UNITED STATES

## Abstract

Maintaining blood-brain barrier integrity and minimizing neuronal injury are critical components of any therapeutic intervention following ischemic stroke. However, a low level of vitamin D hormone is a risk factor for many vascular diseases including stroke. The neuroprotective effects of 1,25(OH)2D3 (vitamin D) after ischemic stroke have been studied, but it is not known whether it prevents ischemic injury to brain endothelial cells, a key component of the neurovascular unit. We analyzed the effect of 1,25(OH)_2_D_3_ on brain endothelial cell barrier integrity and tight junction proteins after hypoxia/reoxygenation in a mouse brain endothelial cell culture model that closely mimics many of the features of the blood-brain barrier *in vitro*. Following hypoxic injury in bEnd.3 cells, 1,25(OH)_2_D_3_ treatment prevented the decrease in barrier function as measured by transendothelial electrical resistance and permeability of FITC-dextran (40 kDa), the decrease in the expression of the tight junction proteins zonula occludin-1, claudin-5, and occludin, the activation of NF—*k*B, and the increase in matrix metalloproteinase-9 expression. These responses were blocked when the interaction of 1,25(OH) )_2_D_3_ with the vitamin D receptor (VDR) was inhibited by pyridoxal 5’-phosphate treatment. Our findings show a direct, VDR-mediated, protective effect of 1,25(OH) )_2_D_3_ against ischemic injury-induced blood-brain barrier dysfunction in cerebral endothelial cells.

## Introduction

Cerebrovascular disorders including ischemic stroke are among the main causes of death and disability in industrialized countries. A large number of studies have shown that cerebral ischemia-reperfusion injury causes structural and functional breakdown of the blood–brain barrier (BBB), resulting in increased BBB permeability [1], and the extent of disruption is directly correlated with the severity and duration of the insult [2]. BBB breakdown not only facilitates brain edema and hemorrhage, but has also been linked to an increase in the expression of various cytokines and chemokines, predisposing the brain to a secondary cascade of ischemic injury. Following a brain injury, rapid protection against BBB disruption would be a critical component of any therapeutic intervention to minimize secondary neuronal injury.

The BBB regulates the transport of molecules to the central nervous system and restricts permeability across brain endothelium [3]. Tight junction (TJ) proteins are the most prominent feature of brain endothelium for maintaining BBB integrity and a critical component of the paracellular pathway, which is vulnerable to ischemic injury [4]. Many studies using *in vitro* BBB models have shown that hypoxia and hypoxia/reoxygenation (H/R) induce an increase in BBB permeability and/or TJ disturbance [5]. Factors leading to BBB disruption during and after stroke include alteration in intracellular calcium, production of vascular endothelial growth factor (VEGF), and increased production of nitric oxide and reactive oxygen species (ROS) [6]. ROS contribute to brain injury by inflicting damage on proteins, lipids, and nucleic acids, as well as by activating a number of redox-sensitive signaling pathways. Many laboratories have shown that the production of ROS influences BBB permeability through the upregulation of cell-disrupting enzymes and protein complexes such as matrix metalloproteinases (MMPs), nuclear factor kappa B (NF-*k*B), VEGF and activator protein 1 (AP-1), among others [7,8].

Vitamin D is a steroid hormone synthesized in human skin from 7-dehydrocholesterol by UV light, and is primarily metabolized in the liver and then in the kidney into calcitriol (1,25(OH)_2_D_3_). Calcitriol is the most biologically active metabolite of vitamin D and an FDA-approved molecule whose neuroprotective and neurotrophic actions are being increasingly recognized, and it may prove to be a candidate intervention for stroke. Calcitriol exerts steroid-like effects throughout the body, directly affecting the expression of multiple genes [9] through the nuclear vitamin D receptor (VDR). While 1,25(OH)_2_D_3_ has classically been associated with systemic calcium homeostasis, there is now substantial evidence that it is also a potent modulator of the immune system that can regulate inflammation, neuromuscular function, cell-cycle control, and neuroprotective functions by regulating neurotrophin production [10]. Low levels of 25-hydroxyvitamin D3 (25(OH)D) and 1,25-dihydroxyvitamin D (1,25(OH)2D) are independently predictive for fatal strokes, while 1,25(OH)_2_D_3_ deficiency exacerbates experimental stroke injury in rats [11]. Although the neuroprotective effects of 1,25(OH)_2_D_3_ after ischemic stroke are well documented, less is known about the effects of vitamin D and its underlying mechanisms on brain endothelial cells after hypoxic injury.

We do know that the actions of 1,25(OH)_2_D_3_ are mediated primarily by the VDR, a member of the nuclear receptor family [12]. Vitamin D has been shown to exert its immuno-modulatory activity on both mononuclear and polynuclear cell lines through its effects on the VDR [13]. The important role of the VDR in mediating the effect of vitamin D has been demonstrated in VDR-knockout mice, in which cardiomyocytes developed cardiac hypertrophy [14] in the face of this depletion.

Using bEnd.3 cells as an *in vitro* BBB model, we investigated the effects of 1,25(OH)_2_D_3_ and the role of the VDR in BBB permeability, cerebral endothelial cell death, and increased production of ROS-induced NF-*k*B—MMP-2/9 expression after H/R. Previous studies have found brain endothelial cells to be a suitable *in vitro* BBB model for studying TJ protein and gene expression, sucrose permeability, transendothelial electrical resistance (TEER), and solute transport [15–18]. This model allowed us to study the mechanisms of action of 1,25(OH)_2_D_3_ by directly testing the effects of the VDR antagonist pyridoxal 5’-phosphate (P5P) (the active form of vitamin B), which in *in vivo* testing could have confounding mechanistic effects independent of the VDR [19,20]. We hypothesized that 1,25(OH)_2_D_3_ blocks NF-*k*B-mediated hypoxia-induced BBB disruption and cerebral endothelial cell death by binding to the VDR, and this beneficial effect is blocked by P5P. Our findings in this study show that the hormonal form of vitamin D can play an important role repairing BBB damaged by stroke.

## Materials and Methods

### Endothelial cell culture

An immortalized mouse brain endothelial cell line (bEnd.3) was purchased from American Type Culture Collection (ATCC, Manassas, VA) and cultured in Dulbecco’s modified Eagle medium (DMEM; ATCC) supplemented with 10% fetal bovine serum (ATCC). Cell cultures were incubated at 37°C in a 5/95% mixture of CO2 and atmospheric air, and the medium was replaced every 2 days (see below for measurement details).

### Drug treatment

Cells were exposed to various concentrations (0, 5, 20, or 200 nmol/L) of 1,25(OH)_2_D_3_ (Sigma-Aldrich, St Louis, MO) for 24 h before H/R. In all cases, the treatments continued throughout the H/R period. 1,25(OH)_2_D_3_ was dissolved in ethanol (Sigma) and further dilutions were made with culture medium. To investigate the specificity of the functional effects of 1,25(OH)_2_D_3_, we blocked the VDR with the VDR antagonist P5P (1 mM), administering it for 24 h before H/R and continuously over the H/R period with or without 1,25(OH)_2_D_3_.

### Normoxia and H/R study

Normoxic cells were transferred into a serum-free medium of glucose-containing (4.5 g/L) phenol red-free DMEM. To measure BBB permeability without endothelial cell death under ischemic conditions *in vitro*, bEnd.3 cells were exposed to 6-h hypoxia and 3-h reoxygenation as previously described [5]. To measure and quantify cerebral endothelial cell death, bEnd.3 cells were subjected to 16-h hypoxia and 10-min reoxygenation. Based on our preliminary parametric testing, we determined that this prolonged hypoxia was necessary for the cell viability assay. In brief, confluent bEnd.3 cells were subjected to an ischemic injury by transferring cultures to glucose-free medium (DMEM without glucose) pre-equilibrated with 95% N_2_ and 5% CO_2_. The oxygen concentration was ≤0.1% as monitored by an oxygen analyzer (Biospherix, Redfield, NY). Reoxygenation was initiated by adding glucose-containing (4.5 g/L) phenol red-free DMEM for 3h at 37° C in 95% air and 5% CO2.

### BBB permeability assay

Monolayer bEnd.3 cells were seeded on the luminal side of the filter (0.4*μ* m pore size; Corning, Lowell, MA) coated with fibronectin (5*μ*g/mL) in 12-well plates. TEER was measured with a millicell ERS-2 volt/ohm meter (Millipore, Billarica, MA). TEERs of cell-free fibronectin-coated filters were subtracted from the measured TEERs shown as Ω x cm^2^ as previously described [5]. Paracellular BBB permeability with TEER measurement was confirmed using FITC-dextran fluorescein. FITC-dextran (40 μL, 1 mg/ml final concentration) (Sigma) was filled to the insert before 3-h reoxygenation. Fifty μL of medium was collected from the lower chamber. The aliquots were diluted to 1 ml with 1x PBS. We transferred 100 μL of each diluted sample into 96-well black plates and measured the fluorescent content for FITC-dextran at 492/520 nm absorption/emission wavelengths.

### Immunofluorescence staining

Monolayer bEnd.3 cells were grown directly on cover slips in 6-well plates (4 x 10^4^; Corning) and cultured for 5 days. After being washed in phosphate-buffered saline (PBS; pH 7.4), the cells were fixed in 4% paraformaldehyde for 10 min at RT. After permeabilization with 0.5% Triton X-100 or 0.1% saponin followed by blocking with 5% bovine albumin (BSA), cells were incubated with anti-ZO-1, anti-claudin-5, anti-occludin (1:200; Invitrogen), or NF-kB p65 (1:200; Cell Signaling, Danvers, MA) in PBS with 5% BSA at 4° C overnight and followed by Alexa fluor 488 conjugated anti-rabbit or Alexa fluor 594 conjugated anti-mouse IgG (1:400; Molecular Probes, Eugene, OR) in 5% BSA for 1h at RT. The cell nuclei were counterstained with DAPI.

### Western blot analysis

Endothelial (bEnd.3) cell homogenate 8–12% was separated in SDS-PAGE gels, transferred to a polyvinylidene difluoride (PVDF) membrane, and incubated with primary antibodies against claudin-5 (1:1000; Invitrogen), occludin (1:1000; Invitrogen), phospho-IkB (1:1000; Cell Signaling), MMP2/9 (1;1000; Abcam, Cambridge, MA), and β-actin (1:3000; Sigma). Enhanced chemiluminescence (Denville Scientific, Metuchen, NJ) was used for visualization. Intensity of the bands was measured using Image Gauge 4.0 (FUJI Film, Tokyo, Japan).

### Reactive oxygen species assays

Fluorescence detection of mitochondrial superoxide and hydrogen peroxide was measured using the fluorogenic probe MitoSOX Red (Invitrogen). MitoSOX Red selectively enters mitochondria within living cells. When oxidized by superoxide anions in the mitochondria, the dye emits red fluorescence. After 60-min hypoxia, cells were switched to reoxygenation conditions and incubated for 20 min at 37° C with MitoSOX Red (5 μmol/L). Cells were then washed with Hanks’ Balanced Salt Solution to remove excess dye. DAPI staining was used to confirm the localization of nucleus to cells. The intensity of MitoSOX Red fluorescence was quantified by ImageJ 1.45s software (NIH). H_2_O_2_ was measured using the HRP-linked Amplex Red (Invitrogen) fluorometric assay. The cell media was reacted with working solution (100 μmol/L Amplex Red reagent and 2 U/mL HRP in a 1X reaction buffer) in 24-well plates and incubated at 37° C for 20 min. Absorbance of the color reaction was determined spectrophotometrically (BioTek, Winooski, VT) at Ex530/Em590. The results were expressed as fluorescence per mg protein.

### MTT assay

For cell viability determination, bEnd.3 cells (2 x 10^5^ cells/mL) were seeded in 96-well plates and subjected to16-h hypoxia and 10 min reoxygenation. Medium containing 5 mg/ml 3-(4,5-dimethylthiazol-2-yl)-2,5-diphenyltetrazolium bromide (MTT) was then added to the cells for a final concentration of 0.5 mg/ml and incubated at 37° C for 4 h. The medium was aspirated, and the formazan product was solubilized with dimethyl sulfoxide. The absorbance of the sample was read directly in the wells at a wavelength of 570 nm. Data were normalized to respective controls and represented as percent viability compared to controls.

### Statistical analysis

Significant differences between treatment groups were determined by one-way analysis of variance (ANOVA) followed by the *post-hoc* Tukey test for independent group comparison using GraphPad Prism 5. Data were expressed as mean ± s.e.m. For all our statistical testing, we considered a *p-*value of <0.05 or less to indicate statistical significance.

## Results

### 1,25(OH)_2_D_3_ prevents BBB disruption via the VDR

**Fig 1:**
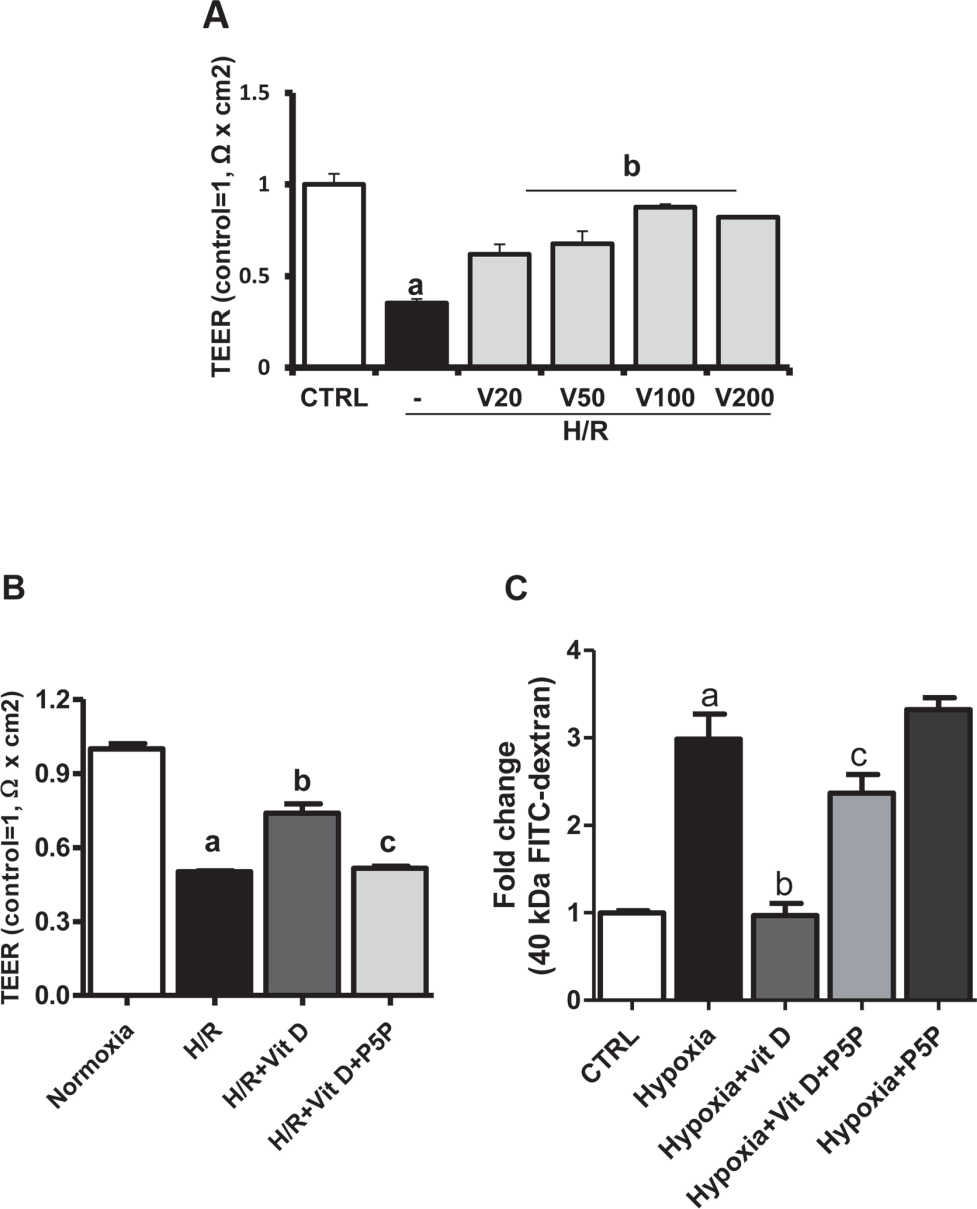
Effects of 1,25(OH)_2_D_3_ on transendothelial electrical resistance (TEER) in bEnd.3 cells under hypoxia/reoxygenation (H/R). **(A)** TEER was decreased by H/R. The decrease was significantly prevented by pretreatment with 1,25(OH)_2_D_3_ (Vit D) at 20, 50, 100, and 200 nM. Values are represented as mean ± s.e.m. **(B-C)** Pretreatment with vitamin D receptor antagonist pyridoxal 5’-phosphate (P5P) decreased the effect of Vit D (with an observed maximum protective dose of 100 nM) on H/R-induced TEER reduction and 40 kDa FITC-dextran increase in bEnd.3 cell monolayers. Values shown are mean ± s.e.m. (n = 3) of a representative experiment. Similar results were obtained from three independent experiments; *p < 0.05, **p < 0.01, and ***p < 0.001.

To test the effects of 1,25(OH)_2_D_3_ on BBB disruption without direct loss of endothelial cells after H/R, we first measured TEER, an important parameter of paracellular BBB permeability in cell cultures. We found that TEER values statistically significantly decreased after 6-h hypoxia/3-h reoxygenation compared with normoxia (Fig. 1). Next we examined the dose-response in TEER values after H/R in bEnd.3 cells pre-treated with 1,25(OH)_2_D_3_ at concentrations of 0, 5, 20, or 200 nM for 24 h before H/R. We found a statistically significant dose-dependent increase, and identified the optimal effective dose of 100 nM (Fig. 1A).

To confirm the effect of 1,25(OH)_2_D_3_ on BBB permeability using TEER measurement, we added 40 kDa FITC-dextran to the upper compartment of a Transwell after 6-h hypoxia and measured the level of dextran across the BBB in the lower chamber after 3-h reoxygenation. The level of 40kDa FITC-dextran was statistically significantly increased after H/R, and blocked by treatment with 1,25(OH)_2_D_3_ (100 nM) (*p* < 0.001, Fig. 1C).

Treatment with P5P blocked the protective effect of 1,25(OH)_2_D_3_ on H/R-induced reduction of TEER values and on the H/R-induced increase of FITC-dextran across the BBB in bEnd.3 cells (*p* < 0.01, Fig. 1B and C), suggesting that the effect of 1,25(OH)_2_D_3_ is VDR-mediated.

### The protective effects of 1,25(OH)_2_D_3_ on TJ alterations were blocked by P5P

**Fig 2:**
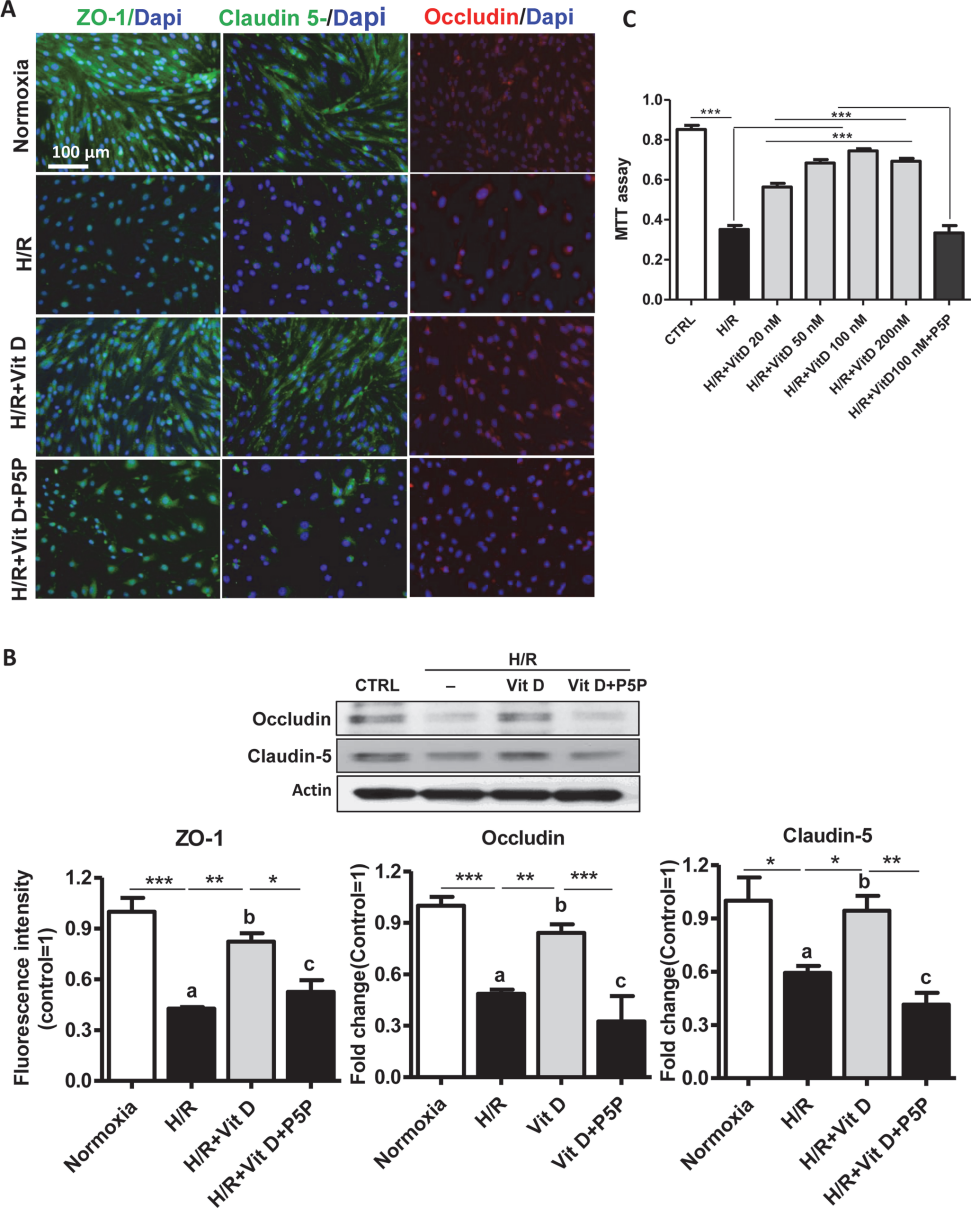
1,25(OH)_2_D_3_ effects on tight junction protein expression and cell viability after hypoxia/reoxygenation in bEnd.3 cells. **(A)** Representative double immunofluorescence staining of ZO-1 (green), claudin-5 (green), occludin (red), and 4’,6-Diamidino-2-phenylindole (DAPI, blue) under hypoxia-reoxygenation (H/R) in the presence or absence of 100 nmol/L 1,25(OH)_2_D_3_ with or without vitamin D receptor antagonist pyridoxal 5 phosphate pyridoxal 5’-phosphate (P5P) in brain endothelial cells. **(B)** Immunoblot images and quantitative data showing the expression of occludin and claudin-5 after H/R in bEnd.3 cell monolayers. H/R-induced loss of occludin and claudin-5 compared with normoxia was prevented by pretreatment with 1,25(OH)_2_D_3_. These effects were blocked by P5P treatment. The density of proteins in normoxia was used as a standard (arbitrary unit) to compare the relative density of the other groups. Values shown are mean ± s.e.m. (n = 3). Similar results were obtained from three independent experiments. **(C)** Representative data from MTT assay showing cell viability after 16 h hypoxia/10 min reperfusion in b.End3 cells. The reduced cell viability after hypoxia was prevented by pretreatment with 1,25(OH)_2_D_3_. These effects were blocked by P5P treatment. Values shown are mean ± s.e.m. (n = 16). * *p* < 0.05, ** *p* < 0.01, and *** *p*< 0.001

Studies have shown that TJ integrity is related to TEER in *in vitro* BBB models [21]. We investigated whether the effect of 1,25(OH)_2_D_3_ on BBB permeability without cerebral endothelial cell death was associated with the TJ proteins ZO-1, claudin-5, or occludin and whether any such effect was mediated through the VDR in H/R-mediated BBB injury. Immunofluorescence staining in bEnd.3 cell monolayers showed that the immunoreactivity of claudin-5, occludin and ZO-1 was decreased and intermittently stained in the cell-to-cell borders in H/R compared with the intact proteins in normoxia. These decreases were prevented with 1,25(OH)_2_D_3_ treatment. Further, the protective effects of 1,25(OH)_2_D_3_ on these TJs were blocked by treatment with the VDR receptor antagonist P5P (Fig. 2A). Western blotting showed that claudin-5 and occludin levels decreased significantly *(p* < 0.001) in bEnd.3 cells after H/R, and this decrease was prevented by 1,25(OH)_2_D_3_ treatment *(p* < 0.01, Fig. 2A and B). Again, the effect of 1,25(OH)_2_D_3_ was blocked by P5P treatment (*p* < 0.001, Fig. 2B). We used an MTT assay to test the effect of 1,25(OH)_2_D_3_ on cell viability after H/R. Cell viability was decreased by H/R (60%, *p* < 0.001), 1,25(OH)_2_D_3_ treatment dose-dependently blocked the decrease (*p* < 0.001, Fig. 2C), and P5P treatment cancelled that effect (*p* < 0.001, Fig. 2C).

### Effects of 1,25(OH)_2_D_3_ on ROS

**Fig 3:**
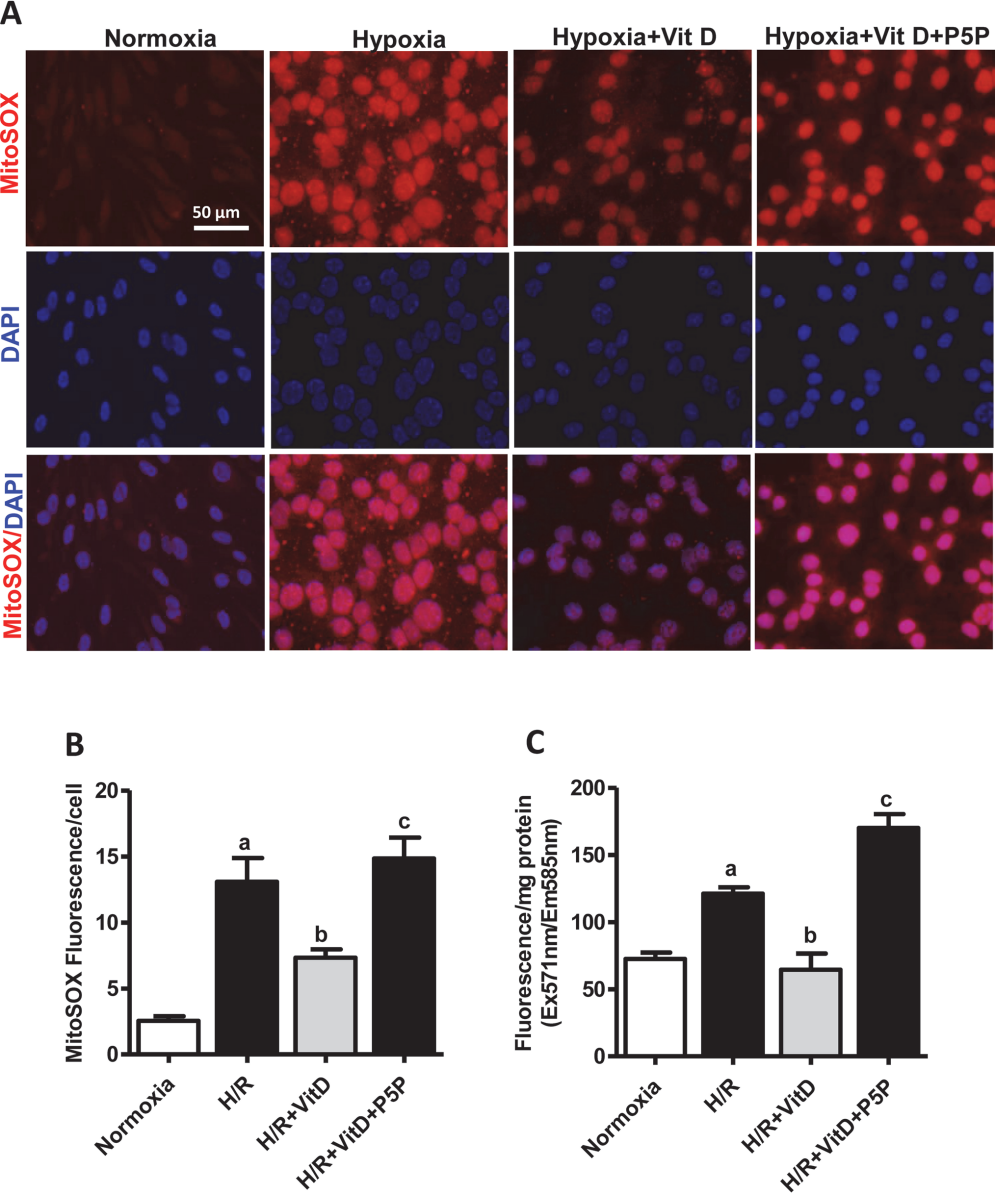
Effects of 1,25(OH)_2_D_3_ on hypoxia/reoxygenation-induced mitochondrial superoxide production and hydrogen peroxide in bEnd.3 cells. **(A)** Mitochondrial superoxide production in live cells was measured by fluorescence microscopy using MitoSOX Red dye. Representative fluorescence images show localization of MitoSOX Red fluorescence and DAPI fluorescence. Scale bar 50 μm. **(B)** MitoSOX Red fluorescence per cell was quantified using ImageJ software. Image data from 51–60 cells per treatment condition were averaged (n = 3). **(C)** Extracellular H_2_O_2_ production with the use of Amplex Red (n = 4). Data are expressed as mean ± s.e.m; *p < 0.05, **p < 0.01, and ***p < 0.001.

To determine whether the protective effect of 1,25(OH)_2_D_3_ on BBB damage occurs through inhibition of ROS generation after H/R, we measured mitochondrial superoxide level and intracellular hydrogen peroxide after H/R using MitoSOX Red fluorescence. As shown in Fig. 3, H/R caused a marked increase in MitoSOX Red fluorescence in vehicle-treated cells compared with normoxia. However, this effect was substantially attenuated (58%) in cells pretreated with 100 nmol/L 1,25(OH)_2_D_3_. In contrast, when cells were treated with P5P in addition to 1,25(OH)_2_D_3_, the protective effect of 1,25(OH)_2_D_3_ was substantially decreased.

We then measured the effect of 1,25(OH)_2_D_3_ on the level of intracellular hydrogen peroxide, superoxide’s downstream product, because of its longer half-life and stability compared to other ROS. H_2_O_2_ generation in bEnd.3 cells was measured by Amplex Red/HRP assay (Fig. 3C). Hypoxia induced a statistically significant increase of H_2_O_2_ production compared with normoxia. Treatment with 1,25(OH)_2_D_3_ elicited statistically significant *(p* < 0.01) inhibition of H_2_O_2_ production in bEnd.3 cells compared to hypoxia alone, whereas treatment with P5P and 1,25(OH)_2_D_3_ statistically significantly *(p* < 0.001) increased the Amplex Red/HRP signals compared to the 1,25(OH)_2_D_3_ probe (Fig. 3C). These data demonstrate that 1,25(OH)_2_D_3_ can reduce the production of ROS in a VDR-dependent manner.

### Effects of 1,25(OH)_2_D_3_ on NF-kB activation

**Fig 4:**
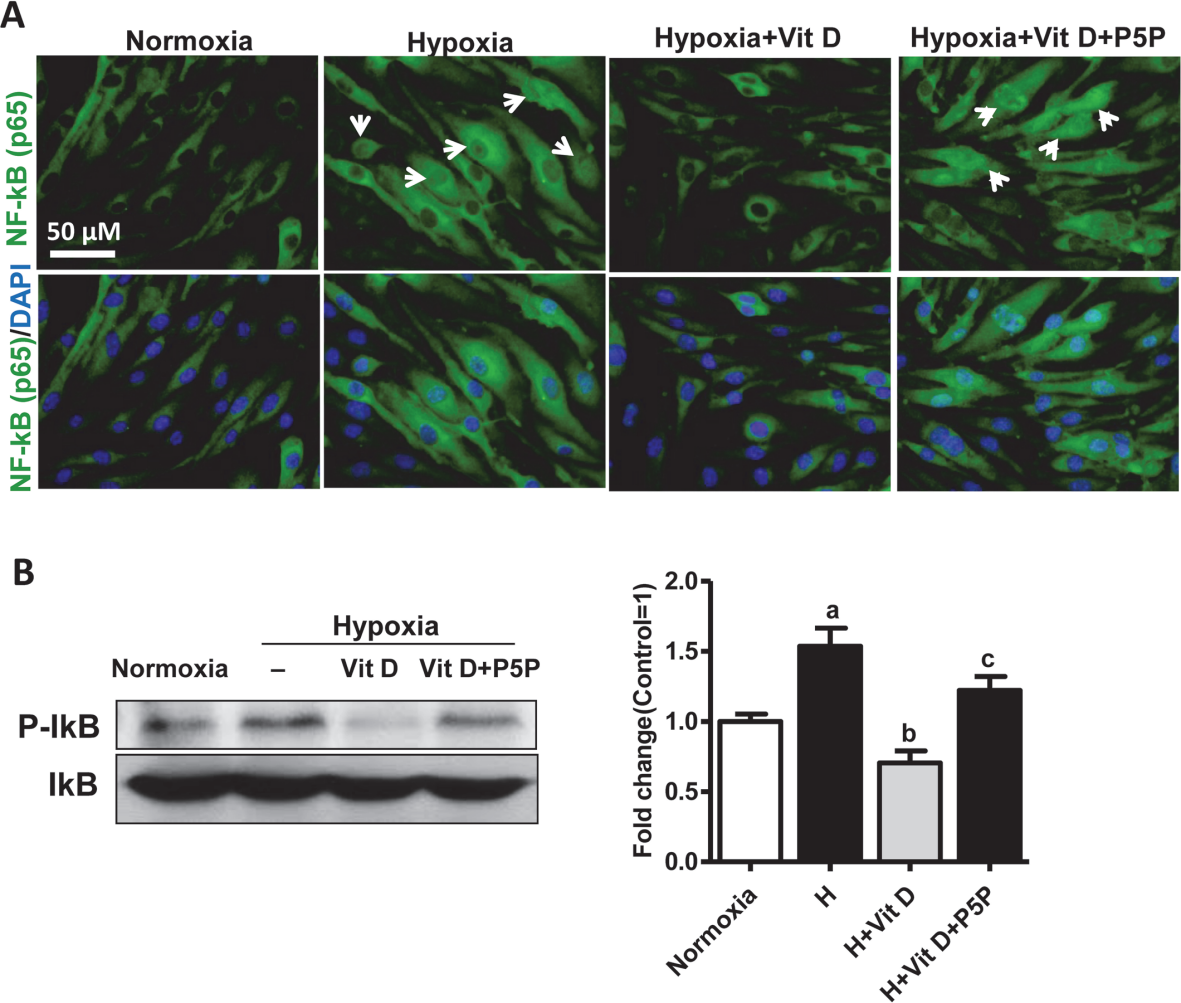
Effect of 1,25(OH)2D3 on NF-kB activation in bEnd.3 cells after hypoxia. **(A)** Representative immunofluorescence images show the translocation of the p65 subunit of NF-κB (green) with DAPI (blue) nuclear counterstaining. bEnd.3 cells were treated with 1,25(OH)_2_D_3_ (100 nmol/L), or 1,25(OH)_2_D_3_ plus vitamin D receptor antagonist pyridoxal 5’-phosphate (P5P; 1 mM) under hypoxia/reoxygenation. Scale bar 50 μm. 1,25(OH)_2_D_3_ prevented the hypoxia-induced translocation of the p65 subunit of NF-kB into the nucleus, which was blocked by P5P treatment. **(B)** Protein levels of phosphorylated IkBα in bEnd.3 cells as determined using Western blotting and imaging analysis. Representative Western blots for IkBα. The density of proteins in normoxia was used as a standard (arbitrary unit) to compare the relative density of the other groups. Values shown are mean ± s.e.m. (n = 3) of a representative experiment. Similar results were obtained from three independent experiments; *p < 0.05, **p < 0.01, and ***p < 0.001.

We assessed the effect of 1,25(OH)_2_D_3_ on the activation of NF-*k*B and the role of the VDR in that effect. We treated bEnd.3 cells with 1,25(OH)_2_D_3_ or 1,25(OH)_2_D_3_ with P5P under hypoxia. We used immunocytochemistry to assess the translocation of p65 after hypoxia. As shown in Fig. 4A, in normoxic cells, p65 staining resided primarily in the cytoplasm, whereas hypoxia induced the translocation of the p65 subunit of NF-*k*B into the nucleus. Treatment with 1,25(OH)_2_D_3_ inhibited this translocation and P5P with 1,25(OH)_2_D_3_ eliminated that effect (Fig. 4A). We also measured the phosphorylation of I*k*B after hypoxia with Western blotting and found that induced phosphorylation of I*k*B was statistically significantly *(p* < 0.01) inhibited by treatment with 1,25(OH)_2_D_3,_ and P5P blocked that effect *(p* < 0.05) (Fig. 4B). These findings demonstrate that the inhibitory effect of 1,25(OH)_2_D_3_ on hypoxia-induced NF-*k*B activation is VDR-mediated.

### Effects of 1,25(OH)_2_D_3_ on MMP-2/9

**Fig 5:**
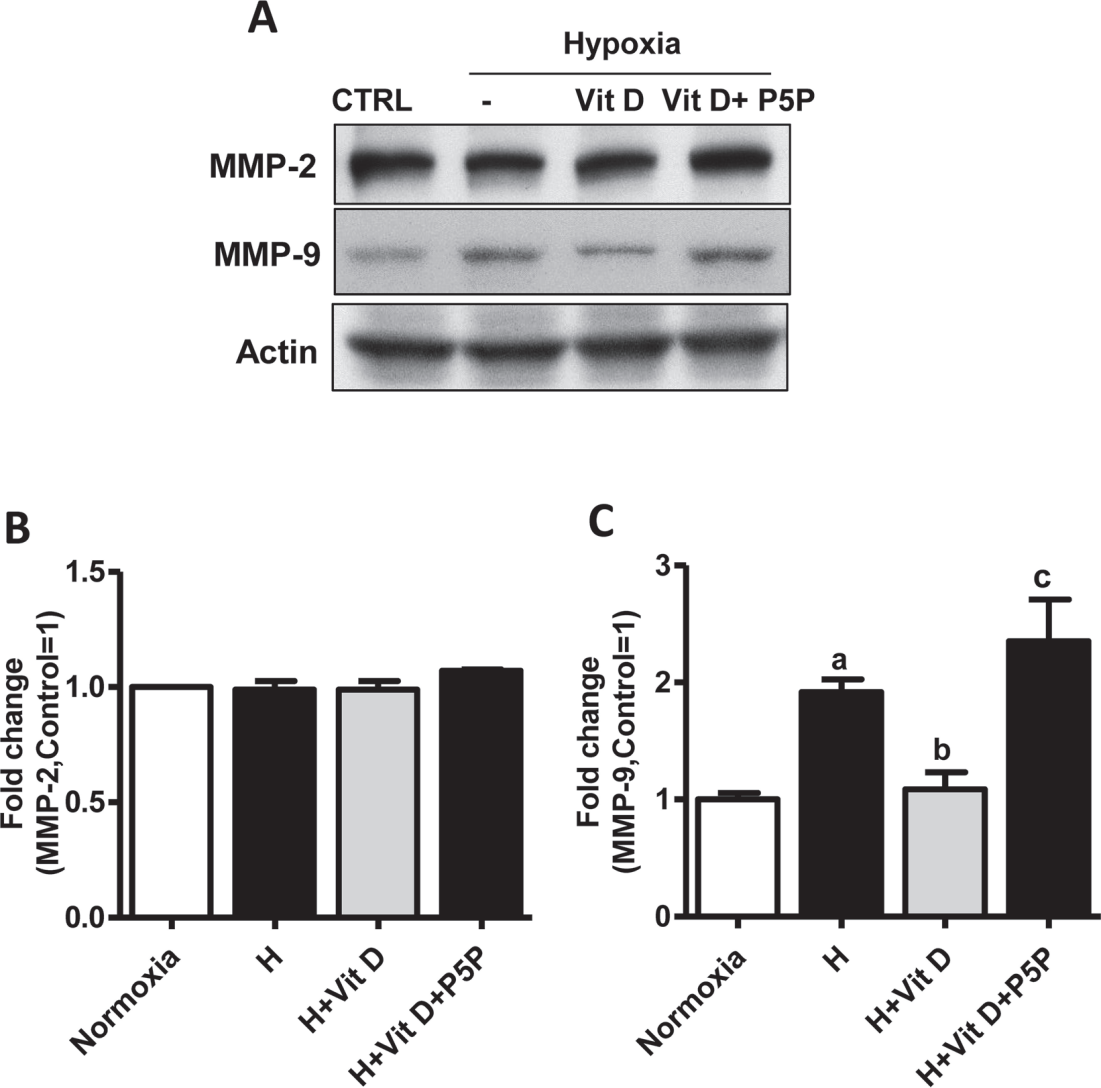
Effects of 1,25(OH)_2_D_3_ on MMP-2/9 in bEnd.3 cells. Cells were pretreated with 1,25(OH)_2_D_3_ at 100 nM or 1,25(OH)_2_D_3_ with vitamin D receptor antagonist pyridoxal-5-phosphate (1mM) for 24 h. **(A)** Representative Western blots for MMP-2/9. **(B-C)** The density of proteins in normoxia was used as a standard (arbitrary unit) to compare the relative density of the other groups. Values shown are mean ± s.e.m. Similar results were obtained from three independent experiments; a, different from normoxia; *p < 0.05, **p < 0.01, and ***p < 0.001.

We tested the possibility that hypoxic injury could affect MMP-2/9 expression in bEnd.3 cells and observed a statistically significant *(p* < 0.05) increase in the expression of MMP-9 but not of MMP-2 after hypoxia. We then evaluated the effect of 1,25(OH)_2_D_3_ on MMP-9 expression in bEnd.3 cells. As shown in Fig. 5, 1,25(OH)_2_D_3_ statistically significantly *(p* < 0.01) decreased MMP-9 expression compared to hypoxia, and P5P treatment blocked this effect *(p* < 0.001). We take these results to indicate that MMP-9 is the major gelatinase contributing to BBB disruption after H/R in bEnd.3 cells, and the inhibitory effect of 1,25(OH)_2_D_3_ on MMP-9 expression is VDR-mediated.

## Discussion

Several lines of evidence show that vitamin D hormone has neuroprotective effects following ischemic brain injury [11], but the role of vitamin D and the VDR in H/R-induced BBB permeability has not yet been investigated. In the present study, we found that 1,25(OH)_2_D_3_ attenuated H/R-induced TJ loss and therefore membrane permeability by blocking the increased production of ROS, NF-*k*B activation, and MMP-9 expression. These protective effects of 1,25(OH)_2_D_3_ were blocked by treatment with the VDR antagonist P5P. The genomic action of 1,25(OH)_2_D_3_, is mediated through the interaction of the VDR with vitamin D response elements (VDRES) from the target genes [22].

To investigate the effect of 1,25(OH)2D3 on BBB function after H/R, we monitored TEER and FITC-dextran permeation, the typical and widely used markers for measuring *in vitro* functionality of BBB TJs and permeability, respectively, in bEnd.3 cells after 6-h hypoxia and 3-h reoxygenation [5,23–25]. In our study, TEER values dropped and FITC-dextran permeation across the BBB increased. We found that adding 1,25(OH)2D3 prevented the decrease of TEER and the increase of 40 kDa FITC-dextran. P5P blocked these effects (Fig. 1B and C), suggesting that they are VDR-mediated. To the best of our knowledge the present study is the first to show the role of the VDR in modulating BBB permeability. Our results suggest that VDR modulation can be targeted as a possible therapeutic intervention for restoring BBB permeability following ischemic insult.

TJs play an important role in preserving BBB integrity under various pathological conditions.

A number of studies have indicated that hypoxia and H/R lead to disruption of BBB TJs in *in vitro* models of the BBB [26]. We and others have previously shown that claudin-5 and occludin expression is highly susceptible during ischemic events and also correlates with TEER changes in brain endothelial cells [5,27]. The literature also reports a reduced level of ZO-1 following hypoxic exposure [28], a finding consistent with our current ZO expression results as measured by immunocytochemistry (Fig. 2A). The observed TJ alterations in our experiment may have been caused by the loss of ZO-1. Because cytoplasmic ZO-1 is linked with actin filaments and the transmembrane protein claudin-5 [29], impairment of the actin filaments may lead to a loss or alteration of ZO-1 and claudin-5. As shown in Fig. 2, the loss of TJ proteins after H/R in bEnd.3 cells was prevented by 1,25(OH)_2_D_3_ treatment, and blocking the VDR by P5P diminished the beneficial effect of 1,25(OH)_2_D_3_. These results can be taken to suggest that this VDR-mediated effect of 1,25(OH)_2_D_3_ is associated with stabilization of the TJ complex.

It has been suggested that BBB disruption can start with focal damage to the vascular wall which then results in endothelial cell apoptosis caused by plasma proteins such as CD8+ T lymphocytes, granzyme B and amyloid-β. Endothelial apoptosis is also induced by cytokines including TGF-β1, platelets and ROS *in vitro* [30,31]. Here we found that H/R in bEnd.3 cells induced a marked decrease in cell viability (apoptosis), and 1,25(OH)_2_D_3_ treatment prevented this effect through its actions on the VDR (Fig. 2C).

Several hypotheses have been proposed to explain the molecular mechanisms of BBB dysfunction through regulation of TJ proteins in hypoxic injury, including ROS generation, oxidative stress, upregulation of MMPs, cytokines and VEGF, and activation of Rho kinase and myosin light chain kinase [32,33]. ROS-inducing oxidative stress is a cellular signaling mediator for various molecules such as glutamate [34], cytokines, and growth factors released from activated glia or microglia [34,35], which in turn have been associated with activation of NF-*k*B [36]. These findings led us to investigate the inhibitory effect of 1,25(OH)_2_D_3_ on H/R–induced mitochondrial superoxide and intracellular hydrogen peroxide. It has been proposed that mitochondrial ROS are a major factor in BBB impairment in ischemia followed by reperfusion [37]. Here, we found that brain endothelial cells exposed to H/R display elevated ROS production and this is decreased by 1,25(OH)_2_D_3_ treatment (Fig. 3). Treatment with P5P and 1,25(OH)_2_D_3_ blocks that beneficial effect.

Hypoxia-induced activation of NF-*k*B observed in various cell types is known to be mediated through the phosphorylation of I*k*B [38]. It has been suggested that the intracellular ROS level regulates NF-*k*B activity, but the molecular mechanism involved remains unclear [39]. In most unstimulated cells, the inactive p65 component of NF-*k*B is retained as dimers in the cytoplasm that are bound to I*k*B proteins. After stimulation, I*k*B proteins are phosphorylated and targeted for degradation *via* the proteasome pathway, allowing the translocation of the p65 component of NF-*k*B to the nucleus. Phosphorylation of IkB in Ser32 and Ser36 of the N-terminal leads to ubiquitination-dependent proteolysis and results in the translocation of NF-kB to the nucleus, followed by the activation of specific target genes [38,40]. Here we demonstrate that 1,25(OH)_2_D_3_ prevents the translocation of p65 into the nucleus and the phosphorylation of I*k*B after H/R in bEnd.3 cells, and that the effect of 1,25(OH)_2_D_3_ on reduction of NF-kB activity is mediated through the VDR (Fig. 4). Our results are in line with the data of Tse et al. showing reduced NF-*k*B transcriptional activity after 1,25(OH)_2_D_3_ treatment in VDR-positive MCF-7 breast cancer cells [41]. Our data indicate that the VDR is required for 1,25(OH)_2_D_3_ action on NF-*k*B, an observation supported by Janjetovic et al., who found that 20-hydroxy-vitamin D_3_ did not affect I*k*Bα mRNA levels in keratinocytes lacking the VDR [42].

Several studies have demonstrated that the proteins regulating TJ proteins such as Rho and MCLK induce the activation of the gelatinases MMP-2/9, which contribute to BBB disruption in animal models of ischemia-reperfusion [43,44]. Increased MMP-2 and/or -9 has also been examined in *in vitro* endothelial cells after hypoxia and H/R [43,44]. MMP-2/9 is mainly produced by injury stimulators such as inflammatory cytokines, serine proteinases, transcriptional factors, and free radicals during reperfusion in ischemic stroke. MMP-2/9 has been shown to mediate BBB disruption by degrading TJ proteins such as occludin and claudin-5 in an animal stroke model and an *in vitro* ischemic model [43–45]. Liu et al. [43], using the selective MMP-2/9 inhibitor SB-3CT or its neutralizing antibodies, showed that MMP-2 released in medium is the major enzyme mediating oxygen glucose deprivation-induced occludin degradation. In contrast, in our study we observed that exposure of the bEnd.3 monolayer to hypoxia for 2 h significantly increased MMP-9 but not MMP-2 expression. This discrepancy may be explained by the fact that we evaluated the expression of MMP-2/9 in the cell population as opposed to the cell media used in the Liu et al. study. MMP-2 is responsible for early BBB disruption, while MMP-9 is involved in brain injury at relatively late stroke stages *in vivo* and *in vitro* [43,46]. Based on the results reported here, we think that the increased membrane permeability after 6-h hypoxia/3-h reoxygenation is caused by increased MMP-9 expression rather than by membrane disruption.

The effects of 1,25(OH)_2_D_3_ on MMP-2/9 expression in bEnd.3 cells after hypoxic injury have not been previously reported. Treatment with 1,25(OH)_2_D_3_ prevented the hypoxia-induced increase of MMP-9 expression. Interestingly, blocking of the VDR with P5P eliminated this effect (Fig. 5). This inhibitory effect of 1,25(OH)_2_D_3_ on MMP-9 expression after hypoxia may be explained by the inhibiting action of 1,25(OH)_2_D_3_ on ROS production and NF-*k*B activation.

Increased vascularization or vascular protection following ischemic/reperfusion injury may be critical to mediate functional recovery, with endothelial cells being the primary cell type responsible for angiogenesis [47]. While the beneficial role of angiogenesis in stroke is recognized [48], there is considerable evidence from the cancer literature suggesting that high doses of 1,25-(OH)2D3 and its analogs inhibit cellular proliferation and angiogenesis and stimulate the apoptotic process [49]. While inhibition of tumor invasiveness and angiogenesis by vitamin D is reported to be mediated by inhibition of MMPs, VEGF, and parathyroid hormone-related protein [50], vitamin D has also been reported to stimulate angiogenesis in endothelial colony-forming cells by increasing VEGF expression and pro-MMP-2 activity [51]. VEGF activation has been shown to be beneficial at a later stage of stroke by enhancing angiogenesis [52,53], but in the acute stage, it is also known to increase microvascular permeability, causing increased edema and hemorrhage [53]. Our study shows that 1,25(OH)_2_D_3_ protects against ischemic injury-induced BBB dysfunction in cerebral endothelial cells by protecting them from ROS and proinflammatory cytokines. The differential effects of vitamin D in cancerous and normal tissue merits further investigation.

In stroke, early disruption of the BBB appears to be a major risk factor for hemorrhagic complications and edema formation induced by treatment with tPA, currently the only FDA-approved stroke drug [1,5,54]. Overall efficacy for tPA reperfusion therapy could be increased and risk-to-benefit ratio reduced by preventing endothelial cell injury and BBB breakdown associated with tPA treatment. Although a more detailed *in vivo* study in stroke models with vitamin D is necessary, our *in vitro* results suggest to us that the neurovascular protective effects of 1,25(OH)_2_D_3_ treatment may prove an important adjunct strategy to improve the safety and efficacy of thrombolytic therapies such as tPA.

**Fig 6:**
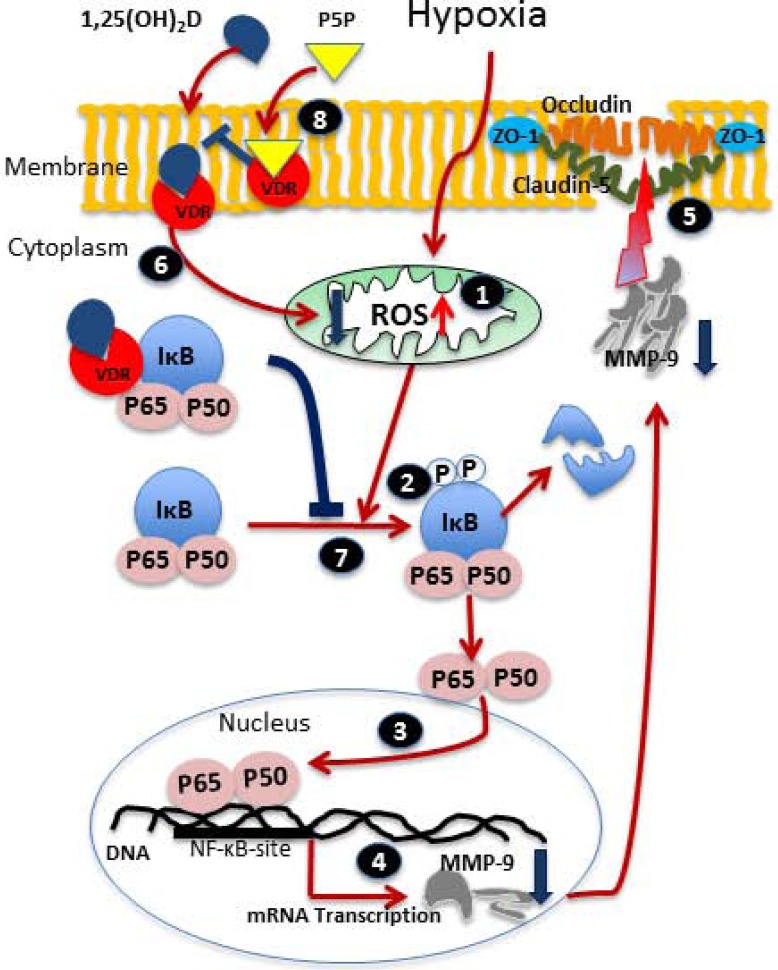
Schematic presentation of a possible mechanism of 1,25(OH)_2_D_3_ action on hypoxia-induced blood-brain barrier (BBB) disruption. Following hypoxic injury, an increase in intracellular reactive oxygen species (ROS) causes mitochondrial dysfunction, which further leads to intracellular oxidative stress (1). As a result, damaged mitochondria activate nuclear factor kappa B (NF-κB) signaling pathways, causing IkB proteosomal degradation *via* phosphorylation of IkBα (2), which leads to proteolysis and the translocation of NF-κB to the nucleus (3). The attachment of NF-κB subunits to its DNA binding site modulates MMP-9 transcription, which leads to increased MMP-9 expression (4). Increased expression of MMP-9 mediates BBB disruption (low TEER values) through degradation of tight junction proteins such as ZO-1, occludin, and claudin-5 (5). 1,25(OH)_2_D_3_ blocks the signaling cascade by binding to the vitamin D receptor (VDR) (6) and preventing the translocation of p65 into the nucleus and the phosphorylation of I*k*B (7). The VDR antagonist pyridoxal 5’-phosphate blocks the effect of 1,25(OH)_2_D_3_ on BBB markers (8).

In conclusion, we found that treatment with 1,25(OH)_2_D_3_ prevented BBB disruption through VDR-dependent mechanisms in cerebral endothelial cells subjected to H/R (Fig. 6). These results show a direct protective effect of 1,25(OH)_2_D_3_ against ischemic injury *in vitro* in cerebral endothelial cells and help to define a novel mechanism for VDR-mediated protection. Given the critical role of endothelial cells for proper brain function, 1,25(OH)_2_D_3_ protection against cerebral endothelial dysfunction by inhibiting ROS production and NF-*k*B activation provides a mechanism to explain the neuroprotective effects of vitamin D hormone.
